# Double-Blinded, Vehicle-Controlled Proof of Concept Study to Investigate the Recurrence of Inflammatory and Noninflammatory Acne Lesions Using Tretinoin Gel (Microsphere) 0.04% in Male Patients after Oral Isotretinoin Use

**DOI:** 10.1155/2012/736532

**Published:** 2012-04-19

**Authors:** Reid Vender, Ronald Vender

**Affiliations:** ^1^Dermatrials Research, 132 Young Street Hamilton, ON, Canada L8N 1V6; ^2^Division of Dermatology, Department of Medicine, McMaster University, Hamilton, ON, Canada L85 4L8

## Abstract

*Background*. Although isotretinoin orally is commonly used for moderate to severe or scarring acne, it is not a cure. Unfortunately recurrence is unpredictable and varies within the acne population. *Objectives*. Using a proof of concept study, determine the recurrence of acne after isotretinoin use in male patients. *Methods*. Twenty males aged 18–45 years old were enrolled. Subjects successfully completed a treatment of acne vulgaris with oral isotretinoin (120–150 mg/kg/course). Subjects were randomized 1 to 1. The study duration was 24 weeks. The primary endpoint measured was the absolute change in lesion counts from baseline to weeks 16 and 24. Local tolerability assessments were measured. *Results*. There were favorable changes in all outcomes measured. Overall, there was a 38.7% lower lesion count with tretinoin 0.04% microsphere gel use versus vehicle. The active product was well tolerated with great patient satisfaction. There were no significant safety issues. The limitations included the low number of patients enrolled, average age, and percentage of patients lost to follow-up. *Conclusion*. In summary, the results favored tretinoin 0.04% microsphere gel in the prevention of recurrent acne after isotretinoin use in male patients over 18 years old over a six-month period.

## 1. Introduction

Acne is a common dermatological disorder that affects millions of North American adolescents and young adults. Although isotretinoin orally is commonly used for moderate to severe or scarring acne, it is not a cure. Even postisotretinoin acne can recur. The use of topical retinoids is part of basic and early acne treatment to treat comedonal noninflammatory acne lesions and occasional inflammatory lesions. After oral isotretinoin, it is not uncommon for comedones as well as noninflammatory and inflammatory papules to recur. Unfortunately this is unpredictable and varies within the acne population. Despite this, there has been no formal study to look at the prevention of recurrence of these acne lesions after isotretinoin. This information may enhance the therapeutic options for postisotretinoin patients in order to prevent recurrence of their disease. The study product was tretinoin 0.04% gel (microsphere) indicated for the topical treatment of acne vulgaris. 

## 2. Materials and Methods

### 2.1. Study Design

This was a double-blinded vehicle controlled proof of concept study with an enrollment of 20 male subjects. Subjects were randomized 1 : 1 to study product or vehicle. Subjects were randomly assigned to each group by an unblinded dispenser. The study duration was 24 weeks with visits at week 0 (baseline), week 4, week 8, week 16, and week 24. All study products were administered once daily (and left on for a minimum of 8 hours) for 24 weeks. Subjects applied a sufficient amount of study product to cover the entire face. The study was approved by an independent ethics review board. The primary objective was to assess the efficacy of tretinoin 0.04% gel (microsphere) on the recurrence rate of acne after isotretinoin use compared to vehicle. The secondary objective was to assess the safety and tolerance of tretinoin 0.04% gel (microsphere) after isotretinoin use.

The following information was collected at each visit in the study: medical history/review of systems, concomitant medications query, adverse events, lesion counts, Investigator's Static Global Assessment (ISGA) (Table 1), and Subject's Global Assessment (SGA) (Table 2), and local tolerability assessments by the investigator (LTA) and subject (SALT) were also determined (Tables 3 and 4). The lesion counts were carried out by a blinded physician (dermatologist) trained and experienced in acne lesion counting and rating. Any missing data for subjects were imputated as intent to treat (iTT) and last observation carried forward (LOCF). Simple statistical methods were used. Poisson 95% Confidence intervals were calculated using the Northwest Public Health Observatory Small Area Database Excel download program. (http://www.nwpho.org.uk/sadb/). The lesion count, ISGA, SGA, LTA, and SALT values were calculated using the mean of all values for that visit for all patients with LOCF.

## 3. Inclusion and Exclusion Criteria

### 3.1. Inclusion Criteria

Subjects were males aged between 18 and 45 years old who had successfully completed a treatment of acne with oral isotretinoin (minimum 4 months/maximum 6 months with an average of 5 months and a total of 120–150 mg/kg/course), could attend scheduled study follow-up visits at the outpatient dermatology clinic, agreed to informed consent for participation in a study, and agreed to comply with the treatment and follow up procedures.

### 3.2. Exclusion Criteria

Subjects meeting any of the following criteria were not be eligible for study admission: subjects who received isotretinoin for conditions other than acne vulgaris, subjects who had been off isotretinoin for less than 30 days (so the immediate beneficial effects of isotretinoin would be mostly gone) or more than 90 days (to try to capture recurrence of acne before the benefits of isotretinoin would be completely gone) at the time of enrollment of the study, subjects who had used prescription topical acne treatment (tretinoin, benzoyl peroxide (BPO), topical antibiotics, or any combination products) between the end of therapy of isotretinoin within 2 weeks of study enrollment or oral antibiotics of any type between the end of therapy of isotretinoin within 4 weeks of study enrollment and throughout the study, subjects who had been previously diagnosed with an endocrinologic disorder likely to cause acne such as genital/adrenal hyperplasia, adrenal tumors, or any other hypoandrogenic state, subjects who are used any systemic medications likely to cause or abate acne such as oral phenytoin or any other epileptic, finasteride, spironolactone, or flutamide, testosterone, or dietary body-building protein powders, and subjects who used topical corticosteroids on the face or systemic corticosteroids within the past 4 weeks. Subjects who used inhaled, intraarticular, or intra-lesional steroids other than for facial acne were acceptable.

Also excluded were subjects who currently used any medication that in the opinion of the investigator could affect the action or evaluation of the study product or place the subject at undue risk. The use of abradants, facials, and peels containing glycolic or other acids; masks, washes, or soaps containing BPO, salicylic acid, or sulfacetamide sodium; nonmild facial abradants, facials, peels containing glycolic or other acids; masks, washes, or soaps containing BPO, salicylic acid, or sulfacetamide sodium; non-mild facial cleansers; and moisturizers that contained retinol, salicylic acid, or *α*- or *β*-hydroxy acids within the past 2 weeks of randomization and throughout the duration of the study was prohibited.

Also prohibited was planned use of medications that are reported to exacerbate acne (e.g., megadoses of certain vitamins, such as vitamin D (>2000 IU per day) and vitamin B12 (>1 mg/day), haloperidol, halogens (e.g., iodide and bromide), lithium, hydantoin, and phenobarbital, because these could also impact efficacy assessments. Subjects who planned to use multivitamins (without vitamin A), iron supplements, and folate were acceptable.

Subjects were excluded that had a facial procedure (such as chemical or laser peel, microdermabrasion, blue light treatment, etc.) performed by an esthetician, beautician, physician, nurse, or other practitioner, within the past 4 weeks or planned to be performed during the conduct of the study. If subjects had a known hypersensitivity or have had previous allergic reaction to any of the active components or excipients of the study product, used any investigational therapy within 4 weeks of randomization, or were currently participating in another clinical study, currently abusing drugs or alcohol (drug screening was not required), had a significant medical history of being immunocompromised, had other conditions that, in the judgment of the investigator, would put the subject at unacceptable risk for participation in the study, had any major illness within 30 days before the screening examination, were current employees of the investigator or sponsor involved in the study, or were an immediate family member (partner, offspring, parents, siblings, or sibling's offspring) of an employee involved in the study, they were also excluded.

## 4. Study Drug Administration

Detailed instructions concerning protocol requirements and application of the study product were provided to the subject. The study products (active and vehicle) were provided in unlabeled grey plastic 50 g pump dispensers. A moisturizer and cleanser provided were the only other products allowed to be used on the face during the duration of the study. The moisturizer was a noncomedogenic, water-based facial moisturizer that was lightweight and nongreasy as well as hypoallergenic, fragrance-free, and alcohol-free (Neutrogena Moisture Oil-Free, Johnson & Johnson, Montreal, Canada). The cleanser was soap-free and alcohol-free as well as noncomedogenic and hypoallergenic (Neutrogena Fresh Foaming Cleanser, Johnson & Johnson, Montreal, Canada). The subject was instructed to wash their face first and allow the areas to fully dry for approximately 20 to 30 minutes before applying study product. Subjects were verbally instructed to apply a sufficient amount (two pea-sized drops) of study product to cover the entire face (including the forehead, nose, cheeks, and chin). Subjects were instructed to consistently apply study product either in the morning or in the evening throughout the 24 weeks of the study.

## 5. Results

The patient's characteristics in the active and vehicle groups were similar as seen in Table 5.

The average lesion counts for the active and vehicle groups are presented in Table 6. Compared to vehicle the active group provided a 38.7% lower lesion count.

Overall, during the study period, the ISGA favored the active ingredient with a lower average grade of acne (a more favorable response) (see Table 7), except at week 24 where no difference was noted.

The SGA correlated well with the ISGA and again seemed to favor the active group (Table 8) with a score two levels lower than that of the vehicle group.

Due to the small number of patients it is difficult to assess the true statistical significance of trend toward the appearance of clinical efficacy of tretinoin compared to vehicle. It seems that one can state that there appears to be a difference.

Overall there were no serious adverse events during the study. There were no adverse events reported during the study either. This may be due to the age of the patients participating in the study that may be reluctant to complain despite direct questioning. Both the active ingredient and vehicle were well tolerated and no treatment was interrupted due to any local tolerability complaints or local side effects. There was only a minimal difference local tolerability score of 0.2 in the vehicle group compared to the active group (range 0–4). The assessments of both the investigator (LTA) and subjects (SALT) correlated well. See Tables 9 and 10.

The total number of missed applications per course of six months of the study averaged 4.4 for the active group (range 0–10) and 3.8 for the vehicle group (range 0–18). For this patient population the averaged missing is only 2.4% and 2.1% of the total possible applications during the six-month study period. Unfortunately three of the active group patients and two of the vehicle group patients were lost to follow-up at various points in the study but their data was imputed as intent to treat with last observation carried forward. This was a loss of a quarter of the patients.

## 6. Discussion

Acne is a common dermatological disorder that peaks at the age of 18 and can affect most adolescents as stated by Nighland and Grossman [1].

Topical retinoids such as tretinoin and adapalene have been clinically proven to be efficacious in mild to moderate cases but severe acne often requires systemic medications such as antibiotics or oral isotretinoin.

Oral isotretinoin has been established to be an effective treatment for severe acne over the past two decades as reported by Wysowski et al. [2]. The recurrence of acne or acne relapse after isotretinoin usage has not been well studied. The prevention of acne after oral isotretinoin has not been studied until this proof of concept study was established. Azoulay et al. [3, 4] reported that a Canadian-nested case-control study of 17,351 first time isotretinoin users revealed that 7100 (41%) of subjects experienced a relapse of acne requiring an antiacne medication (isotretinoin or other). Twenty-six percent of these relapse patients did require a second course of isotretinoin within approximately two years after the end of their first treatment. Twenty percent of the acne relapse patients only required topical treatment. The factors associated with these recurrences were male patients under 16 years of age, living in an urban area and low cumulative dose under 2450 mg over five months. This low-dose was only in 25% of the population-based cohort in the general medical providers as reported by Azoulay et al. [3, 4]. The number of acne relapse patients in the low dose group was not provided.

Layton et al. [5, 6] reported a similar relapse rate of 39% of isotretinoin patients (*n* = 34) with the highest relapsers in those patients receiving a cumulative dose of less than 120 mg/kg per course. The average age was 20.8 years. Twenty-one percent of these patients required topical therapy alone. Seventy-eight percent of the relapse occurred in the first 18 months. Twenty percent of the relapsers were predominantly facial acne. This study studied only facial acne.

Stainforth et al. [7] reported that in their group of 299 patients, 22.7% required repeat courses of treatment. Some of these were for relapse or partial response and a small proportion for psychological reasons. It is not known what the actual relapse rate of acne was as the paper dealt with the factors predicting the need for more than one course of isotretinoin. Their patients had predominantly facial acne.

Therefore reports of acne relapse range from 21 to 41% by Azoulay [3, 4], Layton [5, 6], Chivot [8], White [9], and their coauthors. However there are no reports on the prevention of these potential relapse after isotretinoin. This study was a proof of concept to determine whether acne could be prevented in an adult male population after a single course of isotretinoin. Overall the results favored tretinoin 0.04% microsphere gel in the prevention of recurrent acne compared to vehicle over a period of six months.

Overall there was a trend to the efficacy of tretinoin 0.04% microsphere gel to the prevention of recurrent acne after isotretinoin use in male patients over 18 years old over a six-month period. A 38.7% lower lesion count was observed in the tretinoin 0.04% microsphere gel group. The discrepancy between the lesion count scores and the ISGA assessment at week 24 can be explained by the fact that the ISGA is an overall global assessment and is performed before the lesion counts as not to bias the evaluator.

The active product was well tolerated with great patient satisfaction that correlated well between the investigator's and subject's assessment of tolerability with only 0.2 point difference.

The strengths of this study presented are the facts that the patients were all treated similarly with a course of 120 mg/kg over five months, all were males over eighteen years of age, all were treated by the same dermatologist, and all were from urban areas. The patients had previously not required one or two courses of isotretinoin. 

The weakness of this study is the low number of patients enrolled, the average age of the patients, the percentage of patients lost to follow-up, the male predominance, and possible the short duration of preventative use of the study agent after isotretinoin (six months). Larger and longer follow-up studies are required (ClinicalTrials.Gov Identifier: NCT00939133).

## Figures and Tables

**Table 1:** Investigator's Static Global Assessment.

Investigator's Static Global Assessment	
0: clear skin with no inflammatory or noninflammatory lesions	
1: almost clear: rare noninflammatory lesions with no more than rare papules	
2: mild severity: greater than Grade 1, some noninflammatory lesions with no more than a few inflammatory lesions (papules/pustules only, no nodular lesions)	
3: moderate severity: greater than Grade 2, up to many noninflammatory lesions and may have some inflammatory lesions, but no more than one small nodular lesion	
4: severe: greater than Grade 3, up to many noninflammatory and inflammatory lesions, but no more than a few nodular lesions	
5: very severe: many noninflammatory and inflammatory lesions and more than a few nodular lesions; may have cystic lesions	

**Table 2:** Subject's Global Assessment.

SGA Assessment Scale	
0: my face is basically free of acne, with only an occasional blackhead and/or whitehead	
1: my face has several blackheads and/or whiteheads and small pimples but there are no tender deep-seated bumps or cysts	
2: my face has several to many blackheads and/or whiteheads and small- to medium-sized pimples, and may have one deep-seated bump or cyst	
3: my face has many blackheads and/or whiteheads, many medium- to large-sized pimples, and perhaps a few deep-seated bumps or cysts	
4: my face has blackheads and/or whiteheads, several to many medium- to large-sized pimples, and deep-seated bumps or cysts dominate	

**Table 3:** Local tolerability assessments.

Grade		Subject-assessed itching and burning/stinging
0	None	Normal, no discomfort
1	Slight	A noticeable discomfort that causes intermittent awareness
2	Moderate	A noticeable discomfort that causes intermittent awareness and interferes occasionally with normal daily activities
3	Strong	A definite continuous discomfort that interferes with normal daily activities

**Table 4:** Local tolerability assessments.

Evaluator-assessed erythema, dryness, and peeling				
Grade	Severity	Erythema	Dryness	Peeling
0	Absent	No redness	None	No peeling
1	Slight	Faint red or pink coloration, barely perceptible	Barely perceptible dryness with no flakes or fissure formation	Mild localized peeling
2	Mild	Light red or pink coloration	Easily perceptible dryness with no flakes or fissure formation	Mild and diffuse peeling
3	Moderate	Medium red coloration	Easily noted dryness and flakes but no fissure formation	Moderate and diffuse peeling
4	Severe	Beet red coloration	Easily noted dryness with flakes and fissure formation	Moderate to prominent, dense peeling

**Table 5:** Patient demographics.

Characteristic	Active	Vehicle
Number of subjects (*n*)	10	10
Gender	100% male	100% male
Mean age (range)	21 years old (18–29)	22.4 years old (18–42)
Race	90% Caucasian	80% Caucasian
Duration of acne (range)	5.2 yrs (1–14)	5.6 yrs (2–11)

**Table 6:** Mean lesion counts.

Lesion	Baseline	Week 4	Week 8	Week 16	Week 24
Active (95% CI)	0 (0, 0.369)	0.5 (0, 0.467)	2.1 (0.024, 0.722)	2.1 (0.024, 0.722)	1.2 (0.003, 0.557)
Vehicle (95% CI)	0 (0, 0.369)	1.9 (0.011, 0.642)	2.6 (0.042, 0.801)	2.7 (0.042, 0.801)	3.1 (0.062, 0.877)

**Table 7:** Mean Investigator's Static Global Assessment.

ISGA	Baseline	Week 4	Week 8	Week 16	Week 24
Active (range)	0 (0)	0.1 (0-1)	0.3 (0-1)	0.43 (0-1)	0.33 (0-1)
Vehicle (range)	0 (0)	0.3 (0.1)	0.5 (0-1)	0.43 (0-1)	0.33 (0-1)

**Table 8:** Mean Subject's Global Assessment.

SGA	Baseline	Week 4	Week 8	Week 16	Week 24
Active (Range)	0 (0)	0.13 (0-1)	0.13 (0-1)	0.43 (0-1)	0.13 (0-1)
Vehicle (Range)	0.13 (0-1)	0.33 (0-1)	0.33 (0-1)	0.33 (0-1)	0.33 (0-1)

**Table 9:** Mean Local Tolerability Assessments (LTA) (Investigator).

LTA	Baseline	Week 4	Week 8	Week 16	Week 24
Active (Range)	0 (0)	0.6 (0–3)	0.2 (0–2)	0.4 (0–4)	0.3 (0-1)
Vehicle (Range)	0 (0)	0.1 (0-1)	0.1 (0-1)	0.3 (0–2)	0.2 (0–2)

**Table 10:** Mean Subject's Local Tolerability Assessments (SALT).

SALT	Baseline	Week 4	Week 8	Week 16	Week 24
Active (range)	0 (0)	0.23 (0-1)	0.23 (0-1)	0 (0)	0.13 (0-1)
Vehicle (range)	0 (0)	0 (0)	0 (0)	0 (0)	0 (0)

## References

[1] Nighland M, Grossman R (2008). Tretinoin microsphere gel in facial acne vulgaris: a meta-analysis. *Journal of Drugs in Dermatology*.

[2] Wysowski DK, Swann J, Vega A (2002). Use of isotretinoin (Accutane) in the United States: rapid increase from 1992 through 2000. *Journal of the American Academy of Dermatology*.

[3] Azoulay L, Oraichi D, Bérard A (2007). Isotretinoin therapy and the incidence of acne relapse: a nested case-control study. *British Journal of Dermatology*.

[4] Azoulay L, Bérard A (2009). Isotretinoin therapy and the incidence of acne relapse: a nested case-control study: reply from authors. *British Journal of Dermatology*.

[5] Layton AM, Knaggs H, Taylor J, Cunliffe WJ (1993). Isotretinoin for acne vulgaris—10 years later: a safe and succesful treatment. *British Journal of Dermatology*.

[6] Layton AM, Dréno B, Gollnick H, Mobaken H, Shear N (2009). Isotretinoin therapy and the incidence of acne relapse: a nested case-control study. *British Journal of Dermatology*.

[7] Stainforth JM, Layton AM, Taylor JP, Cunliffe WJ (1993). Isotretinoin for the treatment of acne vulgaris: which factors may predict the need for more than one course?. *British Journal of Dermatology*.

[8] Chivot M, Midoun H (1990). Isotretinoin and acne—a study of relapses. *Dermatologica*.

[9] White GM, Chen W, Yao J, Wolde-Tsadik G (1998). Recurrence rates after the first course of isotretinoin. *Archives of Dermatology*.

